# Central nervous system demyelinating diseases: glial cells at the hub of pathology

**DOI:** 10.3389/fimmu.2023.1135540

**Published:** 2023-05-16

**Authors:** Vinicius Gabriel Coutinho Costa, Sheila Espírito-Santo Araújo, Soniza Vieira Alves-Leon, Flávia Carvalho Alcantara Gomes

**Affiliations:** ^1^ Instituto de Ciências Biomédicas, Universidade Federal do Rio de Janeiro, Rio de Janeiro, Brazil; ^2^ Laboratório de Biologia Celular e Tecidual, Universidade Estadual do Norte Fluminense Darcy Ribeiro, Campos dos Goytacazes, Brazil; ^3^ Hospital Universitário Clementino Fraga Filho, Universidade Federal do Rio de Janeiro, Rio de Janeiro, Brazil; ^4^ Instituto Biomédico, Universidade Federal do Estado do Rio de Janeiro, Rio de Janeiro, Brazil

**Keywords:** demyelinating diseases, astrocyte, glia, neuroinflammation, multiple sclerosis, NMOSD, synaptopathy, disease modifying therapies

## Abstract

Inflammatory demyelinating diseases (IDDs) are among the main causes of inflammatory and neurodegenerative injury of the central nervous system (CNS) in young adult patients. Of these, multiple sclerosis (MS) is the most frequent and studied, as it affects about a million people in the USA alone. The understanding of the mechanisms underlying their pathology has been advancing, although there are still no highly effective disease-modifying treatments for the progressive symptoms and disability in the late stages of disease. Among these mechanisms, the action of glial cells upon lesion and regeneration has become a prominent research topic, helped not only by the discovery of glia as targets of autoantibodies, but also by their role on CNS homeostasis and neuroinflammation. In the present article, we discuss the participation of glial cells in IDDs, as well as their association with demyelination and synaptic dysfunction throughout the course of the disease and in experimental models, with a focus on MS phenotypes. Further, we discuss the involvement of microglia and astrocytes in lesion formation and organization, remyelination, synaptic induction and pruning through different signaling pathways. We argue that evidence of the several glia-mediated mechanisms in the course of CNS demyelinating diseases supports glial cells as viable targets for therapy development.

## 1 Introduction

Demyelinating diseases are mainly characterized by damage to the myelin sheath wrapping the central nervous system (CNS) axons or to oligodendrocytes themselves (1). This large spectrum of diseases produce lesions with similar image findings, but of variable topography, distribution, extension and course. The pathophysiological processes behind the myelin damage is probably different for distinct disorders. These diseases are divided in two categories (2): secondary demyelination, due to subjacent known causes such as infections, malnutrition or deficiency diseases and intoxication; and diseases where demyelination is a primary finding and mechanism. Primary demyelination is associated with a heterogenous spectrum of clinical and pathological phenotypes and, in some cases, with specific biomarkers (3, 4).

The main representative group of these primary demyelinating spectrum disorders are multiple sclerosis (MS), neuromyelitis optica spectrum disorders (NMOSD), acute disseminated encephalomyelitis (ADEM) and myelin oligodendrocyte glycoprotein associated diseases (MOGAD). Other manifestations, such as Marburg disease, Schilder’s disease and Baló’s concentric sclerosis are mostly understood as specific variants of those diseases (5). It is important to note, firstly, the concept of demyelination as a primary feature of disease is an ever-evolving matter. The most glaring example of this concept change is the understanding of aquaporin-4 autoantibody serum-positive NMOSD as an astrocytopathy due to the high involvement of astrocytes in this pathology (3). Viral infection and molecular mimicry, on the other hand, are emerging as strong triggers of demyelinating lesions. The discovery of strong associations between viral infections and proteins with seemingly idiopathic diseases has further blurred the definition of what could be called “primary”. The debate is most clear with the incidence of infection by the Epstein-Barr Virus (EBV) in MS patients (6) or ADEM, MS- and NMOSD-like diseases developing from arbovirus infections (7–9). More recently, with the COVID-19 pandemic, cases of demyelinating lesions following both SARS-CoV-2 infection (10) and vaccination (11) have emerged. According to the discovery of new disease triggers and mechanisms, classification and nomenclature of diseases are therefore subject to eventually become inadequate.

As already stated, the clinical differentiation between these diseases occurs through lesion topography, disease course and, in certain cases, as NMOSD and MOGAD, by a biological biomarker. In MS, the clinical course can vary between the radiologically isolated syndrome (RIS) to the first clinical demyelinating event, the relapsing-remitting or even chronic progression (5, 12). Disease burden also varies greatly. In Marburg disease, considered a fulminant variation of MS, the survival rate rarely surpasses a few months, with relatively more recent cases in literature of people surviving for a few years (14) or even returning to their normal routines (13). On the other hand, progressive phenotypes of MS, often differentiated between primary progressive (PPMS) and secondary progressive (SPMS), may compromise young patients for most of their lives, often resulting in long-time disability and cognitive decline (15). All these phenotypes are now stratified as “active” or “not active” according with the presence of clinical relapsing or new or gadolinium enhanced lesions on magnetic resonance imaging (MRI) during disease course, even on PPMS. This classification approach has been contributing to a clearer scenario of inclusion or exclusion criteria design in clinical trials. NMOSD and MOGAD may also present variable courses, with some patients presenting monophasic disease, while others become recurrent (3). For better synthesis, those diseases main characteristics and what differentiates them in clinical practice are described in **Table 1**.

**Table 1:** Most prevalent forms of CNS inflammatory demyelinating diseases.

Disease	Acute disseminated encephalomyelitis	Myelin Oligodendrocyte Glycoprotein associated disease	Multiple sclerosis	Neuromyelitis optica spectrum disorders
Diagnostic criteria	No established diagnostic criteria consensus. For reference, see: “Acute disseminated encephalomyelitis”, 16.	“Diagnosis of myelin oligodendrocyte glycoprotein antibody-associated disease: International MOGAD Panel proposed criteria”, 17	“Diagnosis of multiple sclerosis: 2017 revisions of the McDonald criteria”, 18	“International consensus diagnostic criteria for neuromyelitis optica spectrum disorders”, 3,
Major criteria	Acute or subacute first-episode encephalitis with focal deficits initiated after an immune challenge.	Presence of a demyelinating event typical for MOGAD and confirmed presence of MOG-IgG. Exclusion of other diagnosis, which must include MS.	Dissemination of disease lesions in time and space, defined by clinical attacks or laboratory and MRI findings in a context where other diagnosis were defined as unlikely. For progressive disease: at least 1 year of disability progression plus suggestive imaging and/or CSF findings.	Presence of a demyelinating event with one clinical core characteristic for NMOSD, associated with confirmed AQP4-IgG. When AQP4-IgG is negative, two core clinical characteristics are needed, corroborated by MRI findings. Exclusion of alternative diagnoses is mandatory.
Clinical course	Fulminant, monophasic.	Monophasic or relapsing- remitting.	Relapsing-remitting; progressively increasing disability, with or without clear relapses.	Relapsing-remitting; rarely monophasic.
MRI findings	Tumefactive white and grey matter lesions on brain T2-weighted MRI; extensive spinal cord lesions with heterogeneous sign. Ring, nodular, gyral or spotty enhancement of lesions.	Longitudinally extensive optic neuritis, with nerve oedema and sheath commitment. Extensive spinal cord lesions, with H pattern and conus involvement. Poorly delimited T2-weighted brain lesions. Leukodystrophy. Leptomeningeal enhancement.	Periventricular, juxtacortical, infratentorial and spinal cord T2-weighted hyperintense lesions, generally with ovoid aspect. Optic neuritis with short optic nerve lesions. T1-weighted hypointense lesions (“black-holes”). “Dawson’s fingers” pattern in corpus callosum. Ring-enhancement of lesions.	Longitudinal extensive traverse myelitis with continuous lesion over 3 or more vertebral segments in T2-weighted image. Long optic nerve lesions, often involving posterior segments and chiasm. Lesions of dorsal medulla, periependymal surfaces of the third and fourth ventricles. Long lesions of the corticospinal tract, corpus callosum and deep white matter.
Epidemiology	Affects both sexes equally; most prevalent in children. Occurs in a scenario of viral illness or vaccination.	Affects both sexes equally. Age has poor impact in incidence, but correlates with clinical presentation.	Affects mostly young adults, women and Caucasian populations. Higher prevalence is correlated with higher latitudes.	Affects both sexes equally. More prevalent in middle aged patients, and in populations of African and Asian descent.
Clinical presentation	Encephalopathy; seizures; focal deficits.	ADEM-like pattern, more common in children; LETM pattern, more common in adults; optic neuritis, often bilateral; sensory and motor focal deficits.	Focal motor and sensory deficits; ataxia; bowel and bladder dysfunction; unilateral optic neuritis.	Area postrema syndrome; severe optic neuritis, with eventual chiasm involvement; focal motor and sensory deficits. Symptoms with poor recovery after relapses.
Biomarkers	There are no currently known specific laboratory findings.	Must have serum MOG-IgG positive in a cell-based assay.	CNS-specific oligoclonal IgG bands in cerebrospinal fluid are present in most cases.	Serum AQP4-IgG positive in a cell-based assay is present in most cases.

Heterogeneity of CNS inflammatory demyelinating diseases (IDDs) is a challenge for developing disease modifying therapies (DMTs). Relapsing remitting multiple sclerosis (RRMS) treatment was the one which mostly evolved in the last two decades, mainly with the use of new immunomodulators, including the use of high efficacy oral drugs, such as fingolimod (19) and cladribine (20). Previously, IDDs of CNS meant an inexorable evolution towards motor disability. Important concepts have been developed since then, arising from the analysis of stratified groups of patients included in the phase III clinical studies. The best outcome, termed no evidence of disease activity (NEDA), is what is intended in the current treatment of CNS IDDs. NEDA-3, the most used NEDA criteria, is characterized by three parameters: absence of flare-ups, absence of progression as measured by Expanded Disability Status Scale (EDSS), and absence of new or active lesions on MRI (21). NEDA-3 status has been used both as an end point (22) and patient selection criteria (23) in clinical trials. It also has been used as a predictive marker for negative disability outcomes (24).

The necessity to further increase NEDA criteria validity and sensitivity led to the adoption of new parameters of disease control. In that regard, NEDA-4, as including annual brain volume within the NEDA-3 criteria (25), was first suggested as a treatment response measure. More recently, a systematic review accounting for 1,000 patients (26) reinforced validity of the NEDA-4 criteria, although comparative analysis showed no advantage over NEDA-3 when predicting long-term disability.

New parameters to better evaluate activity in demyelinating diseases are thus still needed. Among the different NEDA parameter candidates, plasmatic biomarkers for CNS lesion are showing promising results, such as neurofilament light chain (NfL) and glial fibrillary acid protein (GFAP). GFAP in particular is an astrocyte intermediate filament protein, upregulated in conditions of astroglial reactivity. As a biomarker in clinical practice, it has been suggested as a proxy to evaluate neuroinflammation (27).

Although CNS IDDs might differ in several aspects, as previously mentioned, the extensive role performed by glial cells in neuroinflammation is a convergence point among them. This group of cells is often implicated in inflammatory and degenerative disorders of the brain (28). They are a diverse group of non-neuronal cells and are normally responsible for many processes and features of the CNS homeostasis (28), from extracellular matrix composition to synaptic induction and regulation (astrocytes), myelination (oligodendrocytes) and local immune response (microglia). As glial cells perform a huge and diverse functions in CNS development and in the adulthoood, understanding the cellular and molecular mechanisms underlying their functions represent key step to design effective interventions to CNS diseases.

Classically the study of demyelination in disease has focused on oligodendrocytes, as those cells are primarily affected, but recent literature has shown an emerging role of other glial cells, particularly astrocytes and microglia, in regulating not only myelination but also neuroinflammation and neuroplasticity in the course of the disease (29–31).

As different mechanisms at a cellular level may be the cornerstone of developing more accurate biomarkers and effective DMTs, it is imperative to study the different influences which glial cells may exert in primary demyelinating phenotypes. Therefore, the scope of our article is to summarize what is known about the features and pathophysiology of the most prevalent IDDs and to explore the implication of glia in neuroinflammation, demyelination, neurodegeneration and clinical treatment throughout the course of disease. Finally, here we argue that glial cells are viable targets for therapy design and we propose ways in which the study of these cells could lead to new approaches in dealing with CNS demyelinating diseases.

## 2 The processes behind demyelination

Demyelination, as the name suggests, is the pathological hallmark and central disease process in inflammatory demyelinating disorders. Although mechanisms and lesion patterns vary between different diseases, there are a few common denominators essential to inflammatory demyelination. The destruction of myelin sheaths, reducing axons stability and integrity, is present in all of the IDDs per definition, and may be present in both white and grey matters.

Demyelination *per se* is not only a deficit found in cell function, as it may occur naturally as part of the myelin turnover. The disruption of mechanisms such as oligodendrocyte maturation and migration, myelin phagocytosis, myelin sheath renewal and the inflammation itself are consonant with the full pathological progression of demyelination. To understand those dysfunctions, discussed below is both how oligodendrocytes behave in natural myelination and how glial cells interact in its disruption.

### 2.1 Oligodendrocytes and myelination of the CNS

Oligodendrocytes are glial cells derived from the subventricular zone, and are specifically responsible for the myelination process in the CNS. Oligodendrocytes diverge in morphology, function and development among regions. One example, cells in white matter are generally derived from classic Olig-2+ oligodendrocyte progenitor cells (OPC), while grey matter has a higher incidence of NG2+ OPCs (known as NG2 glia) (32).

Differentiation of OPCs into fully myelinating oligodendrocytes is related to both axonal and glial inducing factors. Briefly, immature OPCs emit their endfeet toward axons. The endfeet eventually develop into stable contacts between glial and axonal membranes. Those contacts start to wrap around the axon in a concentric fashion, with the now mature oligodendroglia locally modifying the composition of the membrane, favoring an accumulation of myelin-specific proteins, proteolipids and cholesterol. The myelin membrane, with its composition modified anew, suffers compacting as membrane layers associate between themselves like a zipper (33).

The myelin sheaths perform multiple structural and functional roles on the CNS. Although myelin may be seen in some invertebrates such as crustaceans, the vertebrate CNS myelin is a *sui generis* phenomenon among animals as its morphology and cell origin are unique to that clade (34). Myelin sheaths in vertebrate CNS not only insulate axonal processes, but also confer stability to the axon, control the metabolic pathways of that segment, and are also responsible for limiting neuronal plasticity, through the signaling with the so-called inhibitory proteins. Considering this, the myelin internodes generated by oligodendrocytes can be treated as a unique structure among animals.

The particularities of myelination in CNS are not restricted to myelin itself, though. Of note, different from their peripheral nervous system counterparts, Schwann cells, oligodendrocytes are capable of generating and sustaining multiple myelin internodes at the same time. Another particularity is that, while the myelin sheath itself is the main oligodendroglial feature, these cells are also known to guarantee axonal enlargement and stability during post-natal development through the release of neurotrophic factors (35). This can be considered a natural evolutive advantage, as it permits in humans, for example, a greater neuronal and synaptic plasticity in pre-natal and early post-natal life, both necessary for cognitive, sensitive and motor development.

**Figure 1:**
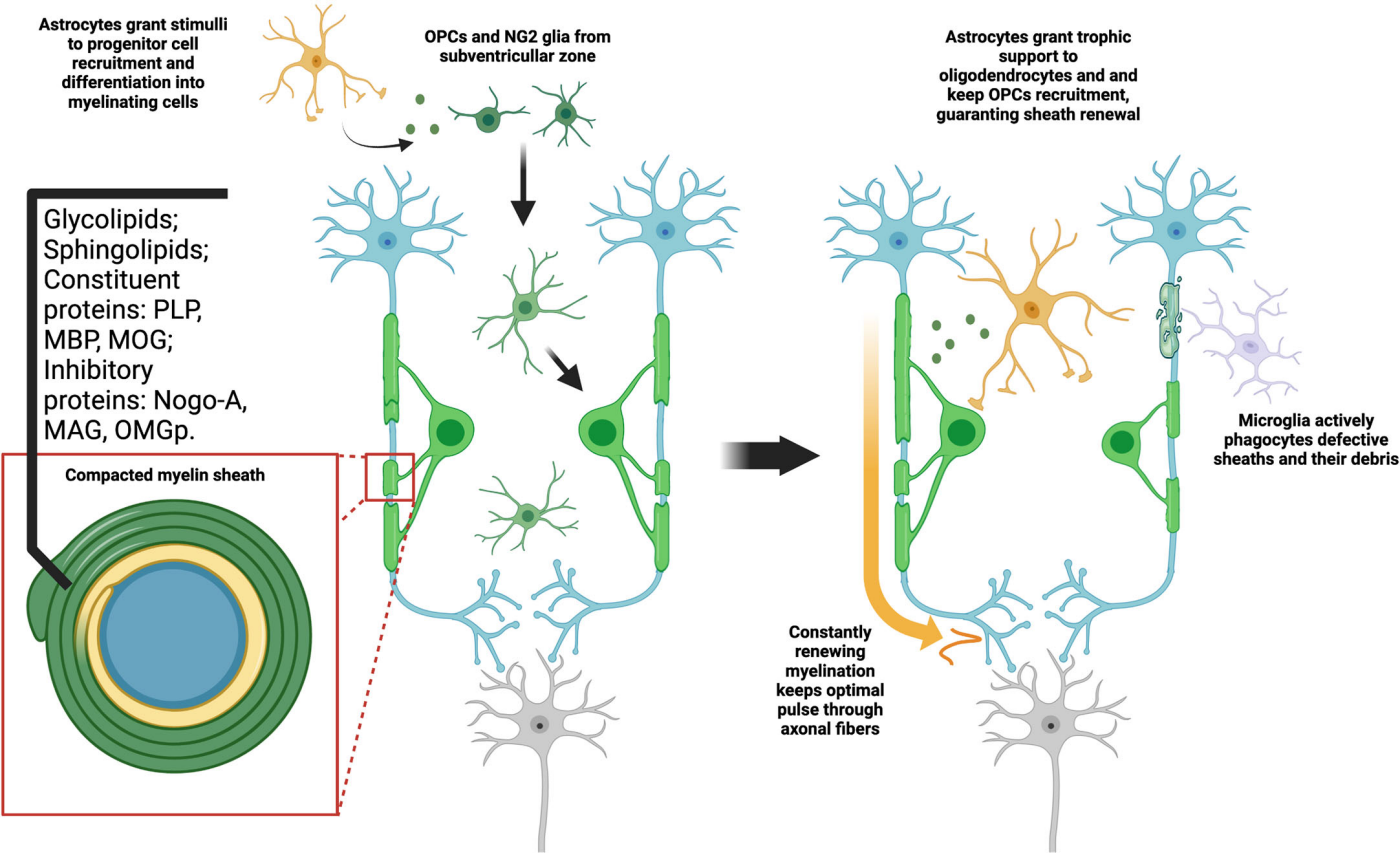
Myelination is a constant process dependent on glial interaction. Both NG2 and OPC types of progenitor cells are capable of differentiating in oligodendrocytes. This process is dependent on signals from other cell types, mainly astrocytes. As they fully mature, they fold a highly compact and specialized cell membrane around axons, originating the myelin sheaths. Aside from the high lipid content, myelin may also provide axon support and signal through a range of typical proteins. As oligodendrocytes maintain high metabolic activity, constantly producing myelin, microglia phagocytes both defective or old sheaths and their debris in the extracellular matrix. At the same time, astrocytes give trophic support to oligodendrocytes and immature OPCs, stimulating formation of new sheaths. This constitutes a tightly adjusted balance, which may be understood as a type of myelin plasticity. The optimal disposition of sheaths along the axon guarantee, thus, not only mechanical stability, but also proper electric insulation and consequent higher pulse velocity.

The characteristics of CNS myelin may also be attributed to their molecular composition, as can be seen in **Figure 1**. Mammalian CNS myelin is noteworthy not only for the proteins responsible for its structure and composition, but also for its myelin-specific proteins which have the role of signaling to other cells of the CNS. Among these myelin-specific proteins, we can highlight the so-called inhibitory proteins: myelin associated glycoprotein (MAG), oligodendrocyte myelin glycoprotein (OMGp) and Nogo-A. These proteins induce response both through RhoA and mTOR pathways, in neurons, astrocytes and microglia. While they are associated with oligodendrocyte maturation and formation of the myelin sheath (36), their action on neurons is associated with growth-cone collapse (37). To them also is attributed the ability to confer circuitry stabilization after the greater part of the post-natal synaptogenesis. In astrocytes, our group demonstrated Nogo-A signaling may limit astroglial control over synaptic formation and function (31). The problem arises that these mechanisms, although essential for CNS development and survival, may be obstacles to both remyelination and axonal regeneration in disease contexts. As different complex pathways intercede, there is no widespread medical strategy to overcome this problem yet. We will later focus on these pathways and interceding limitations.

While oligodendrocytes are the main cell type involved in myelination, it is important to stress that both microglia and astrocytes regulate the production and renewal of myelin. As the myelin sheath is a dynamic structure in constant production, older segments of myelin must be destroyed to avoid formation of defective sheaths or pathological deposits of myelin debris in the extracellular matrix. Microglia, as the primal phagocytic cells of the CNS, are the main elements responsible for this event. Astrocytes, on the other hand, grant trophic support, release factors which stimulate both differentiation of OPCs into oligodendrocytes, and may induce recruitment and migration of those cells in events of remyelination. The roles of these cells may still overlap, as there are cases of astrocytes phagocyting myelin, and microglia seems to be necessary in some cases of non-developmental oligodendrocyte maturation (38, 39). The most essential problem now is to understand that the “plasticity of myelin”, as a current turnover phenomenon, seems not to be solely dependent on the oligodendrocytes (40).

### 2.2 Myelin destruction and neurodegeneration

The central event in the CNS, related to IDDs, is the disruption of myelin turnover within parenchymal lesions. The underlying pathological event varies between disorders and its pathology is not totally clear. In NMOSD, this event is thought to be related to inflammatory reaction after blood-brain barrier (BBB) disruption. In MOGAD it may be caused by direct destruction of myelin by anti-MOG autoantibodies-induced autoimmune reaction. Animal models may also help to understand these triggers. While the experimental autoimmune encephalomyelitis (EAE) model is closer to anti-MOG disease in etiology, cuprizone-induced demyelination relies directly on oligodendrocyte death to induce the disruption. In MS, it’s thought that both BBB disruption, oligodendrocyte death and myelin sheath direct degeneration are present. While none of these are totally representative of the disease, all are useful and competent approximations of its underlying mechanisms.

As far as current evidence from animal models show, axons and internodes are destroyed in disease, while myelin starts to accumulate in the extracellular space. Microglia recruitment happens and primary neuroinflammation elapses, but these cells are insufficient to keep up with pathology. Astrocytes start to also phagocyte myelin debris and isolate active lesions, taking on a “giant astrocyte” reactive profile. The giant astrocytes still induce differentiation of OPCs into fully myelinating oligodendrocytes to compensate for demyelination, but these cells aren’t fully capable of migrating into lesion areas. Active lesions maintain this pattern of partial regeneration until episode remission, when, finally, regeneration overcomes myelin destruction. Once-demyelinated areas may still present themselves as scarred tissue.

In the case of progressive phenotypes of MS, always-active lesions are associated with a disordered circuitry remodeling, due to unstable neurites, and grey matter atrophy, both leading to poor clinical outcomes. In this situation, inflammation does not act as an acute insult. Neurodegeneration becomes a central process instead. With lesions remaining chronically active, remyelination becomes more prominent as a compensatory phenomenon, although dysfunctional. The deposition of myelin debris in extracellular matrix is more extensive, and may limit recovery by reduction of neuronal plasticity. Pathological forms of remyelination may also occur, with the apparition of concentric sheaths and non-compact myelin in axons. Another problem is the varying impact of inflammatory demyelination in the different regions of the CNS. While acute cuprizone intoxication in rodents has shown to lead to increased synaptic density in superficial cortical layers, in the subcortical area, such as the dorsal lateral geniculate nucleus, the contrary has been observed. Demyelinating lesions presented gliosis with lesser density of excitatory synapses, while GABAergic circuits prevail (41).

Nevertheless, what becomes undeniable is the impact of both glial and grey matter disease in MS. When looking into the clinical history of PPMS, some works (42, 43) show that not only grey matter destruction is highly-prevalent, but it also occurs in somewhat related anatomical patterns, which, in turn, strongly correlate with clinical disabilities such as motor impairment and cognitive decline. Cognitive decline itself, while historically considered a secondary phenomenon in demyelinating diseases, has gained more attention recently. As acute disease and relapses are better managed with optimized clinical criteria and therapy, progressive cases (particularly secondary progressive ones) may arise in occurrence and become a greater concern.

The impacts of synaptic dysfunction in MS and other IDDs has led our group to study particularly the effect of myelin proteins in synaptopathy. Synaptopathy, as described in MS, occurs from an imbalance between formation/activity of excitatory (mainly glutamatergic) and inhibitory (GABAergic) synapses. As a result of such dysfunction, excitotoxicity ensues (44). Multiple studies have shown both in MS patients and in the EAE model that inflammatory infiltration of the CNS and cytokine release is directly correlated to synaptic loss in regions such as the hippocampus, the striatum and the neocortex. Uncoupling of the glial and neuronal mechanisms of synaptic homeostasis, such as physiological pruning and neurotransmitter recycling, may be a central link to synaptopathy. For further details, we recommend an extensive review by Georgia Mandelosi and associates (45). It is clear that these dysfunctions, then, are an overlooked facet of IDDs. Clinical trials are currently underway to study the impact of glutamatergic modulating drugs in MS, especially in progressive diseases (46–48), with mixed results.

### 2.3 Myelin inhibitory proteins in synaptopathy

Among potential links for the pathophysiological findings in MS and its animal models, we pay special attention to myelin inhibitory proteins. These are a series of proteins characteristically expressed in the myelin membrane with the potential to limit axonal and synaptic plasticity. This class was first studied and proposed based on its prototypical member, the neurite outgrowth inhibitor (Nogo-A). First described in 2000 by the group of Martin Schwab (37), Nogo-A is a member of the reticulon family. Mainly expressed by oligodendrocytes, it’s a transmembrane protein associated with the myelin sheath, presenting two biologically active residues. The Nogo-66 domain has a canonical receptor in the form of NgR1 (Nogo Receptor 1; 49). Its activation induces a cascade mainly based on RhoA-GTP, which modulates the polymerization of the actin cytoskeleton and ultimately impedes neurite and growth cone formation. The other portion, the N-terminal Nogo-Delta20, is implicated in cell survival, but the understanding of its mechanism is still rudimentary (50). Throughout the last two decades, other proteins have become implicated in these pathways, such as MAG and OMGp, although Nogo-A has remained the most relevant in experimental and clinical investigation.

While Nogo-A signaling is mainly associated to myelination and inhibition of synaptic and axonal plasticity in adult mammalian CNS, it has also been found to correlate with numerous homeostatic and pathological mechanisms, such as found in schizophrenia (51), stroke (52), beta-amyloid disease (53), spinal cord injury (54) and amyotrophic lateral sclerosis (55, 56).

**Figure 2:**
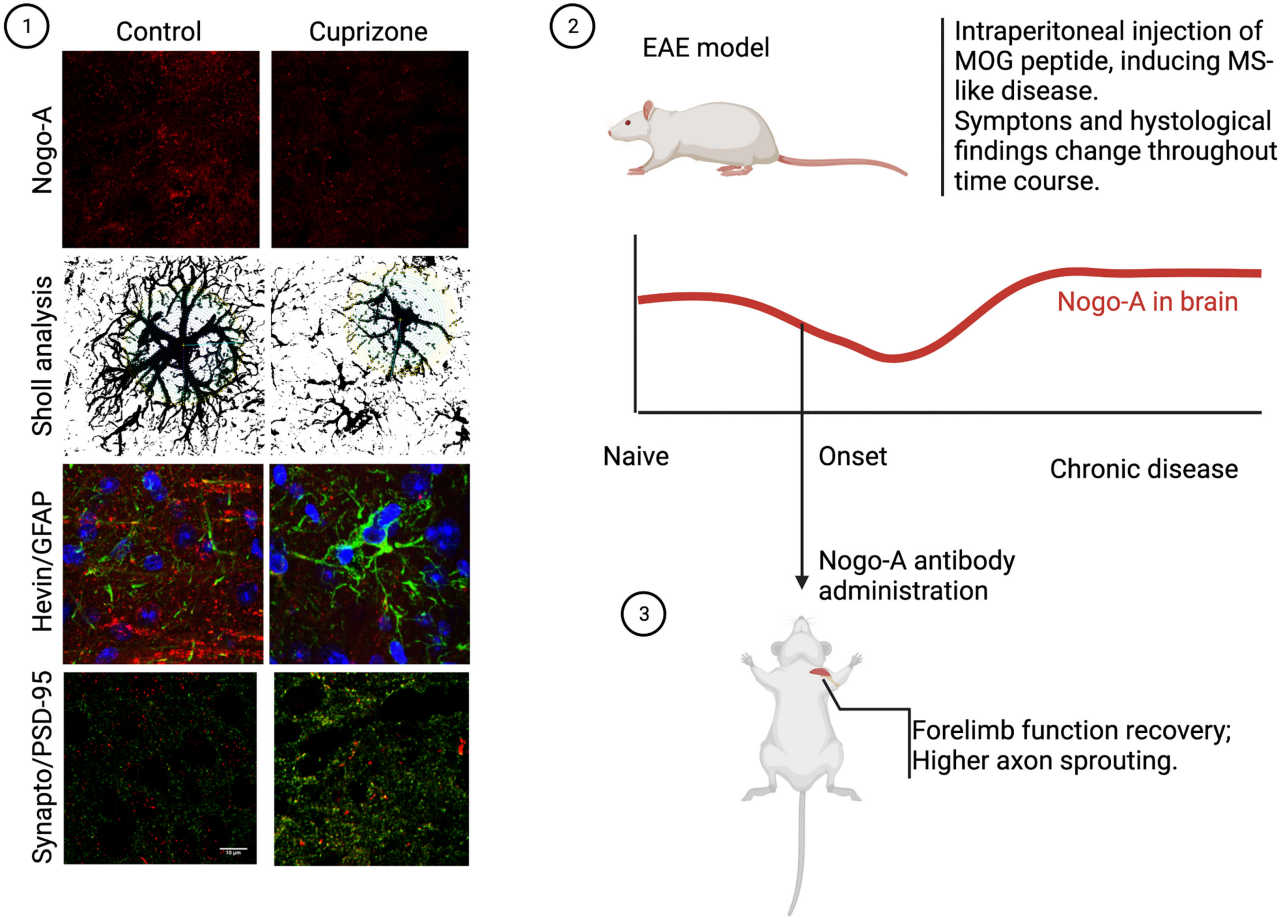
Nogo-A in CNS demyelination models. In our work, we discovered acute demyelination by cuprizone intoxication, leading to almost elimination of cortical Nogo-A, changed both astrocyte morphology and synaptogenic profile. This was also accompanied by higher excitatory synapse density, as can be seen in the photomicrographs above (1). On the other hand, studies of the chronic phase of the EAE model, where myelin debris in lesions play a significant role, Nogo-A was found to be more expressed than baseline (2). In this case, though, a work by Ineichen et al. (58) showed that if early anti-Nogo-A therapy was employed, rapid clinical recovery ensued, with documented tract regrowth (3).

On animal models for MS, NgR1 expression in B cells has been associated with cell recruitment and inflammation, as well as higher myelin immunoreactivity (57). The administration of monoclonal antibodies against Nogo-A had favorable results in the experimental scenario, as it rescued myelination and induced axonal repair in EAE rats (58). Not only histological findings were present, but treatment also brought recovery to forelimb function, as shown in **Figure 2**. Curiously, inactivation of NgR-class receptors in the immune system, particularly CD4+ T cells, showed no effect, which may suggest that Nogo-A plays a minor role in peripheral cell activity, or that it may be almost fully dependent on a non-canonical receptor. Inactivation of S1PR2, for example, a sphingosine receptor which also binds to Nogo-A preventing axonal growth, resulted in increased remyelination in both lysolecithin-induced demyelination and EAE mice (59), further exemplifying the potential role of S1P and its analogues.

**Figure 3:**
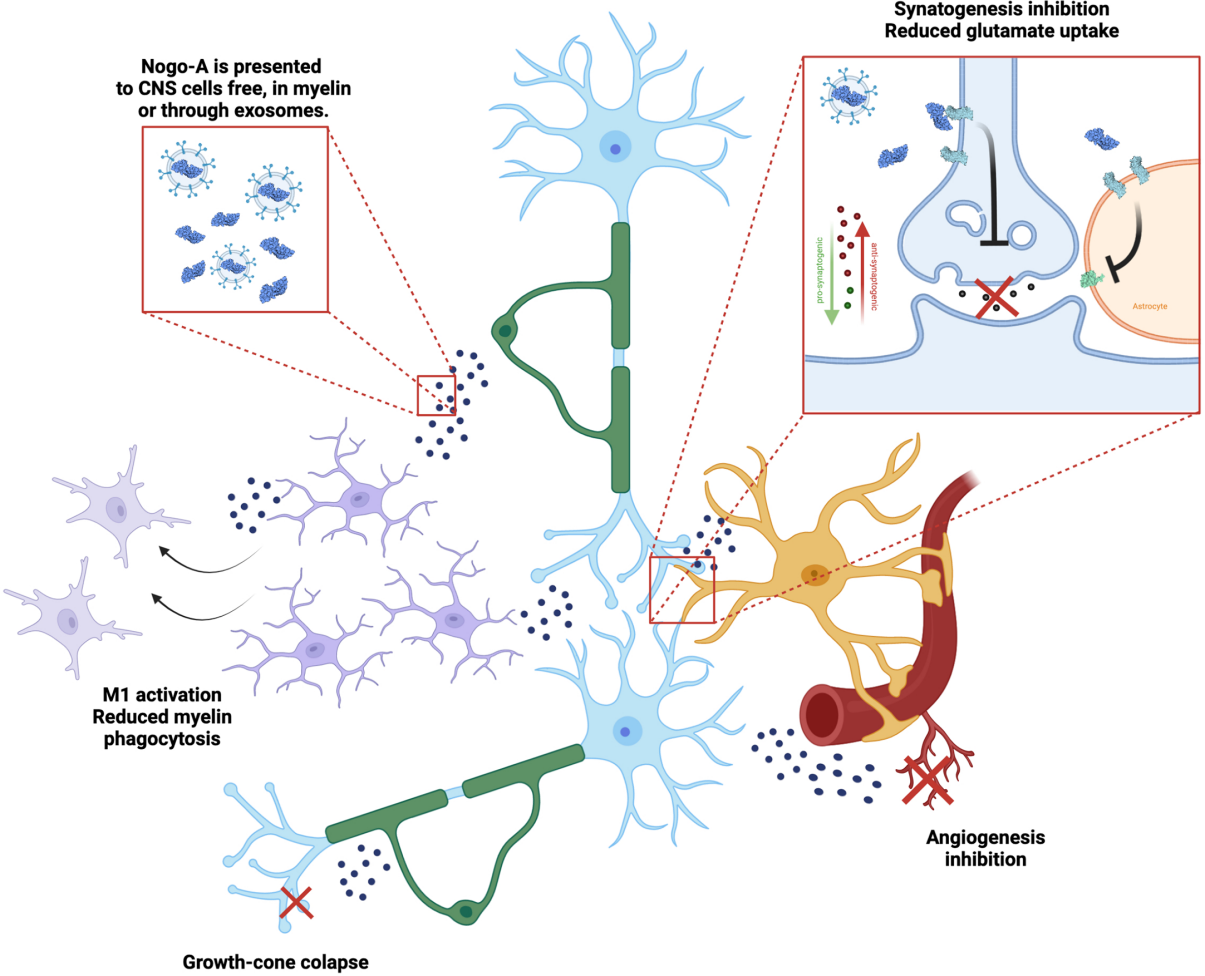
Nogo-A effects on the neuro-glial unit. Nogo-A is a transmembrane protein present in neurons (blue) and oligodendrocytes (green), enriched in myelin sheath. It is presented to different cells in the CNS, while included in the myelin itself, in free debris and also in exosomes. Nogo-A activates mainly the Nogo-66 Receptor 1 (NgR1, light blue), which leads to different effects, such as: microglial (purple) activation and control of phagocytosis, axon growth cone collapse, modulation of astrocytes’ (yellow) synaptogenic profile and glutamate uptake through EEAT transporters (light green), as well as inhibiting angiogenesis (red).

As myelin debris accumulate in the extracellular matrix and anomalous remyelination occurs, it has been suggested that demyelinating chronic disease may be associated with Nogo-A deposition and increased signaling. In a recent article, our research group reported that Nogo-A could also interact with astrocytes, giving them a more antisynaptogenic profile, impairing their mechanisms of synaptic regulation, such as glutamate uptake (31). When Nogo-A was depleted from the visual cortex of mice by acute cuprizone treatment, astrocytes released a higher concentration of synaptogenic factors and excitatory synapses proliferated (**Figure 2**). Although we have not evaluated the case of microglia, previous articles have shown that these cells actively respond to Nogo-A, modulating functions such as cell migration, focal adhesion and inflammation (60, 61), while Nogo-A may impair microglial phagocytic capacity (62). We understand this is further evidence that myelin inhibitory proteins, such as Nogo-A, may serve as a converging point between inflammation, synaptopathy and glial cells in demyelinating diseases, as is described in **Figure 3**. As previously stated, Siponimod, which is a RhoA-GTP inhibitor (63), interestingly has astrocyte-dependent neuroprotective effects, through upregulating glutamate transporters and downregulating inflammatory response to astrocytes (64). Using these pathways for the development of new therapeutic strategies may be a rewarding challenge, as they seem to intervene in domains not yet covered by available treatment alternatives.

## 3 Glial cells and neuroinflammation in demyelinating diseases

Neuroinflammation is a robust hallmark of demyelinating diseases. Although etiologic hypothesis for most of those diseases relies on autoimmunity, there is a complex and interactive system regulating inflammatory processes. While T and B lymphocytes may exert a role in initiation and perpetuation of inflammatory response (65, 66), resident microglia, astrocytes and oligodendrocytes are highly involved in BBB dissolution (67), lesion development, synaptopathy, cytokine release and antigen presentation (68). More recently, a role has also been found for the gut microbiome, which may stimulate T cell function in multiple sclerosis (69).

Whether inflammatory response in demyelination is causal or consequential is still a matter of discussion. In the next section, the mechanisms underlying this process will be discussed.

### 3.1 Peripheral immune response in demyelinating processes

The participation of peripheral immune cells in demyelination pathogenesis was first described in ADEM, the firstly identified IDD of CNS. Early pathological studies already showed ADEM to be characterized by perivascular demyelination associated with inflammatory infiltrates of peripheral immune cells, mainly macrophages, T and B cells (70). The later discovery of similar infiltrates in different phenotypes of MS, and the presence of specific anti-AQP4 and anti-MOG autoantibodies, in NMOSD and MOGAD, both highlighted the participation of peripheral immune cells in demyelination pathogenesis.

Autoimmunity, even if not the only disease trigger, plays a large role in the production of autoantibodies and cytokine release. Genetic predisposition to MS through expression of specific human leukocyte antigen (HLA) alleles associated both with antigen presentation and other autoimmune diseases is also widely documented (71).

Evidence obtained both through clinical studies and pre-clinical research with the EAE model suggest adaptive peripheral immune activity is exerted mainly through CD4+ Th-1 and Th-17 lymphocytes, in a B-cell antigen-presentation dependent manner. B-cells are also important cytokine releasers (72), and producers of oligoclonal bands found within the cerebrospinal fluid in MS (73). Among the identified autoantibodies produced in MS cases, currently only the anti-MOG IgG has been identified as clinically relevant, given that anti-MOG disease has emerged as a new clinical entity altogether (74, 75). CD4+ T-cells, on the other hand, make up the majority of immune cells present within deep demyelinating areas, where they produce primarily IFN-γ and IL-17. While IFN-γ may enhance the antigen-presenting activity of the other cell types, IL-17 facilitates cell infiltration through the BBB (76). Once in the CNS parenchyma, CD4+ T-cells induce inflammatory response from both macrophages and the resident CNS glia: mainly astrocytes and microglia. Finally, CD8+ T cells are also present and affect later phases of inflammatory insult, directly targeting myelin (71, 76).

**Figure 4:**
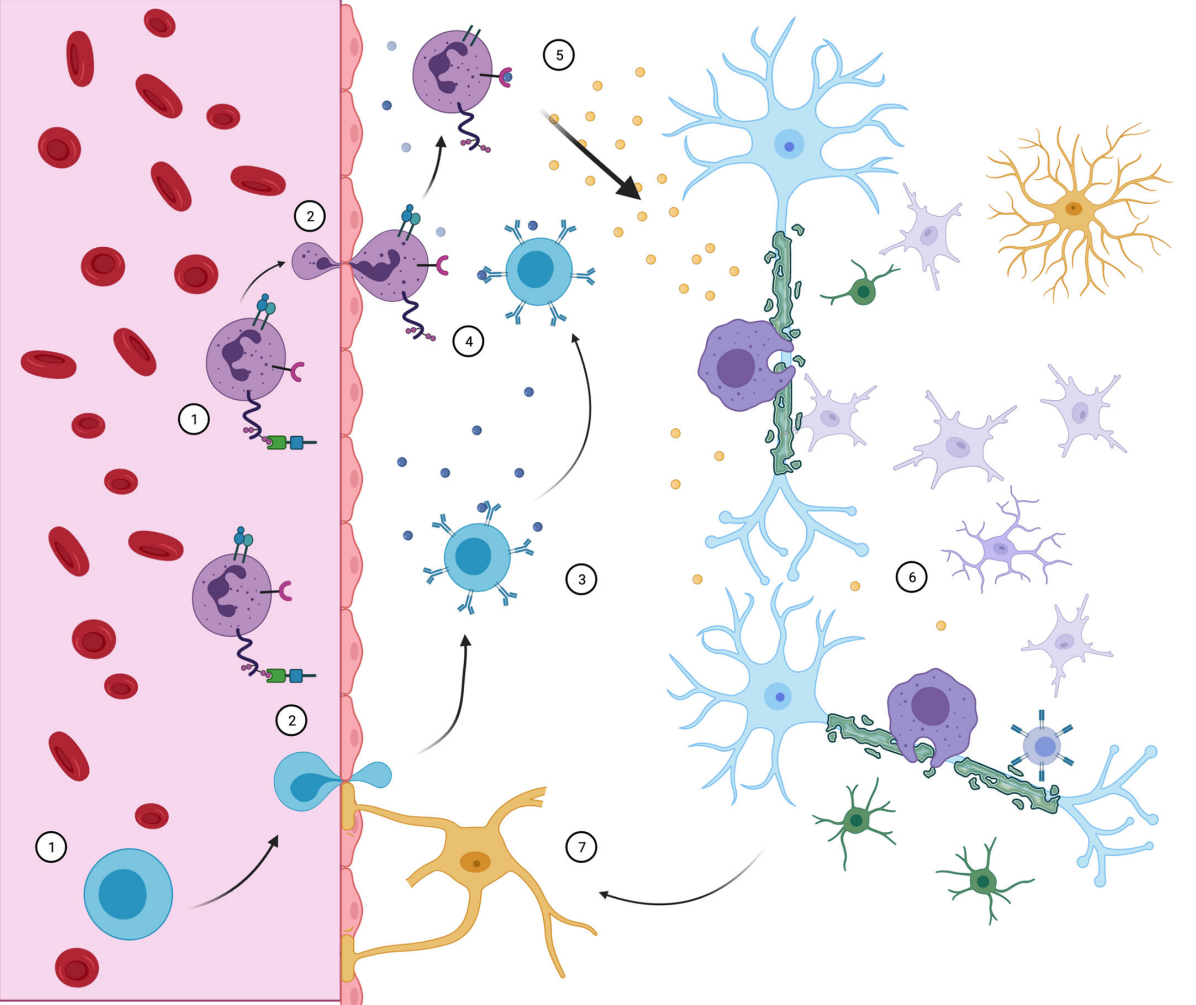
Neuroimmune interaction in MS. CD4+ Th cells (purple) and B cells (blue) access the CNS through the BBB. There, B cells may contact myelin-specific antigens (3) and present them to Th cells (4), triggering inflammatory response (5). With the release of pro- inflammatory cytokines, resident microglia, macrophage, CD8 cells and astrocytes are recruited and develop demyelinating lesions (6), while oligodendrocyte precursors are incapable of compensating for sheath destruction. Peripheral immune cell infiltration maintains itself by further BBB destruction associated with reactive astrocytes (7). Although all events complement themselves in lesion pathophysiology, the order and triggering events are still objects of debate.

In general lines, CNS invasion by these populations may initiate and sustain demyelinating lesions, but is not a sufficient mechanism to explain their dynamics alone. A crosstalk between the different peripheral immune cells and the glia residing in CNS, as showed in **Figure 4**, is necessary for sustaining inflammation and demyelination within lesions. A framework which incorporates those roles may prove more suitable.

### 3.2 Glial cells and their role on CNS homeostasis

Astrocytes are the most numerous cell type of the CNS glia. This group was initially defined for its morphology and understood merely as a secondary component of histoarchitecture. These cells are classically divided between those considered protoplasmatic and those considered fibrous, by their grey and white matter origins, respectively. The common consensus for years was that the astrocytes were homogenous within these groups. Consensus now states that these cells are highly diverse in function and transcriptome pattern among different brain regions (77, 78). The complexity and diversity of astroglia discovered in mammals and, particularly, hominids, has been proposed as a main evolutive step in information processing capacity of the CNS (79).

Astroglial cells form a group of functional units inside the CNS integrating synapses, the neurovascular unit and different circuitries. Astrocytes may communicate between themselves through Ca2+ signaling in gap-junctions, which may synchronize the functions of distinct brain regions. In a way, astrocyte signaling has already been included in some simulation models of neural networks (80). During development and maturity, astrocytes also regulate neuron metabolism through release of gliotransmitters, transport of nutrients and other bioactive molecules through the BBB, regulation of extracellular ion content, CNS organogenesis and control of synapse plasticity and activity (81). Recently, a key role has been attributed to astrocytes during CNS aging (82). The regulation of synapsis specifically is considered one of the central roles of these cells, occurring through a variety of mechanisms. Neurotransmitter uptake and recycling, especially glutamate, may occur directly from the synapse, thus forming a tripartite synapse model, where an astrocyte endfoot remains as an essential third component. Induction of both synaptogenesis and synaptic pruning occur through the release of soluble factors and extracellular matrix molecules, such as TGF-Beta1 (83, 84), Hevin, Glypican-4 and -6, Thrombospondin and SPARC. Another mechanism is that the astrocytes themselves may induce or reinforce synapses by contact-dependent processes. As cells highly active in the homeostasis of the CNS microenvironment, astrocytes have already been shown to contribute to the pathophysiology of neuroinflammatory and neurodegenerative disorders, such as Alzheimer’s (85) and Parkinson’s diseases (86).

Microglia, differently, act mainly as phagocytic cells. They are the resident macrophages of the CNS, composing roughly 15% of the glial cells. Mammalian microglia derive directly from the yolk sac, and populate the CNS prior even to vasculogenesis (87). Although stationary cells, they have highly motile processes, scanning the surrounding environment for insult detection. Although microglia are most known by their activation into anti- or pro-inflammatory states after exposition to injury, these cells retain their homeostatic function, such as inducing neuron survival through IGF-1 secretion. They also act in synaptic remodeling, clearance of metabolites and debris from the extracellular space, and myelin sheath renewal, being responsible for the phagocytosis of old, degenerated sheaths (88).

Lastly, the oligodendrocytes are the main cells implicated in demyelinating disease. While microglia are the main cells linked to physiological myelin phagocytosis, and astrocytes to pathologic phagocytosis in sclerosis plaques, a feature which will be discussed later, oligodendrocytes are the generators of CNS myelin. They originate from OPCs (oligodendrocyte progenitor cells), which, when in an advanced stage of maturation, migrate to the periphery of the axonal fibers and, through a combination of epigenetic, translational and environmental cues interconnected through a SOX10 mediation, differentiate into full myelinating oligodendrocytes (32), which compose roughly 5 to 10% of the glia. Those cells then, with highly active metabolism, produce a compact cytosolic membrane rich in lipids and a particular set of proteins, including MAG, MOG, OMGp, Nogo-A and others. This membrane is overproduced and wrapped around axonal processes at regular intervals, in a continuously-producing fashion. As highly-specialized and metabolic active cells, oligodendrocytes are subject to a high risk of injury through mechanisms such as excitotoxic damage, oxidative stress and inflammatory events (89).

Under the physiological conditions of the CNS, the glial cells converge to facilitate an efficient synaptic signaling, maintaining a plastic circuitry, clean intercellular space and accelerating pulse conduction with dynamic renewal of the myelin sheath. The next section discusses how disruption of those functions by demyelination and central inflammation may implicate in the pathology of IDDs and loss of function. We will shed light in specific signaling pathways underlying glia dysfunction in demyelinating diseases.

### 3.3 Astrocytes in demyelinating conditions

The most direct example of astrocyte involvement in demyelinating events is NMOSD, particularly the AQP4-IgG positive cases. As aquaporin-4 is mainly localized in the astrocyte endfeet, these are the primarily affected cells in this disease. In that manner, NMOSD could also be called an astrocytopathy, along with GFAP-IgG autoimmune encephalitis (90). Current hypothesis asserts that the targeting of astrocytes, and particularly astrocytic endfeet, destroys specific areas of cell-cell interaction, resulting in dissolution of the BBB, neuroinflammation and, finally, demyelinating lesions in the CNS. In this case, demyelination in NMOSD can be understood as a later product of astrocytic inflammatory cues and macrophage infiltration with complement deposition, being one stage of a greater lesional process centered on astroglia (91).

Accordingly, AQP4-IgG was demonstrated to induce IL-6 production and release by astrocytes through the NFkB pathway (92). IL-6, aside from its proinflammatory cytokine activity, may also cause an increase in BBB permeability, contributing to its disruption and further immune cell infiltration in CNS (93). The use of animal models is needed to elucidate other direct roles AQP4-IgG may have on astrocytes. While complement deposition is still understood as a central mechanism, it was recently demonstrated that induction of optic neuritis by human AQP4ab in mice is dependent on Interferon type I signaling (94). Other processes involved include ATP release inducing neuropathic pain (95), internalization of glutamate transporters and activation of other cell types (96).

While not considered a direct target of disease as in NMOSD, astrocytes retain central roles in other forms of demyelination. Initially known by forming glial scar after the initial inflammatory insult, many other functions are now identified for astrocytes in MS such as recruiting leukocytes for CNS, microglia activation, boosting neurodegeneration, as well as contributing for neuroprotection and limiting the area of inflammation (97). They display a reactivity pattern marked by the expression of molecules such as nestin, embryonic neural cell adhesion molecule, FGFr4, EGFr and nerve growth factor (98).

Reactive astrocytes are characteristic of MS lesions, being expanded at the margins of active lesions, adjacent to healthy tissue, and also in the center of chronic ones, in the form of glial scar (99, 100). Support for the contribution of astrocytes in lesion formation has been provided by evidences that reactive astrocytes are observed in the asymptomatic and early phases of the disease, even before a significant inflammatory infiltrate (101, 102). Furthermore, hypertrophic astrocytes containing myelin debris can be seen at the edges of early MS lesions associated with active NFkB pathway, which is not seen in control, myelin-negative astrocytes. In addition, myelin phagocytosis by these cells *in vitro* induces NFkB activation and chemokines expression, associated to the recruitment of leukocytes and microglia (99). Together, these findings indicate that astroglia has the potential to trigger its own activation after myelin phagocytosis and stimulate the inflammatory process in the MS-forming lesion.

In order to more clearly investigate the beneficial or deleterious role of astrocytes in the pathogenesis of MS, studies using genetic or pharmacological approaches have revealed a dichotomous behavior in a disease phase dependent manner. Astrocyte depletion in the acute phase of MS increases disease severity and CNS inflammation (103–105). These findings were corroborated by the study in which genetic depletion of astrocytes from cuprizone-induced demyelination model resulted in impaired microglia recruitment and consequent delay in the clearance of myelin debris and in remyelination (106). It is also possible that the negative effects of depletion in the acute phase are related to the impairment of the BBB (97). On the other hand, astrocyte depletion in the chronic phase of MS resulted in disease attenuation and reduced inflammatory infiltrate (107). Indeed, a specific population of astrocytes, named astrocyte-inflamed in MS (AIMS), has been found to be enriched on the edge of the active chronic MS lesion (108). These cells were shown to be enriched in transcripts for response to injury and corticosteroids, in addition to C3, a marker of neurotoxic astrocytes. This role in the progressive phase deserves even more attention when considering that at this stage of the disease, BBB breakdown is less prominent and neurodegenerative processes are robust and driven by micro- and astroglia (97).

In MS, astrocyte reactivity and its neurotoxic effects have been associated with signaling induced by inflammatory cytokines, free radicals, Toll-like receptor agonists, phagocyted myelin, among others (109). Although there are so many drivers of the astrocyte hypertrophic profile in MS, the mechanisms activated by the majority of them seem to be triggered by neuroinflammation and neurodegeneration. Consonant with this idea, it was shown that although homeostatic astrocytes have heterogeneous signatures in healthy individuals according to the CNS region, when they become reactive in MS, they undergo overlapping activation of pathways, mainly related to cell stress and immune activation (110).

NFkB pathway corresponds to one of the main pathways activated in astrocytes in MS and in models of inflammatory demyelination, and is associated with the production of proinflammatory cytokines and leukocyte recruiting chemokines (109). A recent study with astrocytes exposed to cerebrospinal fluid from a patient with MS revealed that the reactive state of the astrocyte and its neurotoxic effects correlate with the degree of NFkB activation and the inflammatory environment. Thus, astrocytes exposed to cerebrospinal fluid with high but not low inflammation, and in an NFkB pathway-dependent manner, produce a secretome that induces synaptopathy and neuronal injury (111). Furthermore, it is possible that the altered astrocytic NFkB gene has an impact on susceptibility to MS, which until now was closely related to dysfunctions in immune system cells. The study demonstrated that human astrocytes carrying the risk variant rs7665090G, located close to the NFkB gene, show increased signaling of this transcription factor, with higher expression of its target genes, and worsening the pathogenesis of MS. *In vitro* and in MS lesions, there is increased expression of adhesion molecules and chemokines in astrocytes, leading to increased recruitment of lymphocytes and enlargement of lesion size (100). Consistently, downregulation or inactivation of the astrocytic NFkB pathway in EAE model reduces inflammation and improves tissue injury and clinical impairment (100).

Abnormalities in the sphingolipid metabolism pathway may also contribute to changes in the reactive state of astrocytes, contributing to their pathogenicity in MS in an NFkB-dependent manner (109). Lactosylceramide (LacCer), a ceramide-derived sphingolipid, and the enzyme that catalyzes its synthesis (B4GALT6), are overexpressed in the non-obese diabetic EAE model, which mimics the progressive phenotype of MS; and in MS chronic lesions (109). LacCer pathway induce a reactive pro-inflammatory state of astrocytes (112), and *via* CCL2 and GMCSF production induces recruitment and activation of microglia and macrophages. In turn, suppression of LacCer synthesis inhibits resident innate immunity of the CNS and neurodegeneration in the EAE, in addition to inhibiting the activation of human astrocytes (107). Additional studies have shown that LacCer activates cytosolic phospholipase A2 (PLA2) and MAV2 in astrocytes and that, autocrinely, this pathway leads to NFkB activation and transcription of inflammatory factors, contributing to the pathogenesis of EAE and MS. The interaction between MAV2 and PLA2 displaces MAV2 from its HEK partner, the latter two involved in lactate synthesis (113). Thus, in addition to leading to inflammation, the pathway triggered by LacCer also contributes to neurodegeneration, by impairing metabolic support for neurons through reduced lactate levels.

Sphingosine-1-phosphate (S1P) pathway also seems to be upregulated in astrocytes in active and chronic MS lesions through an increase in the S1P receptors, S1P1 and S1P3 (114). The S1P pathway is involved with the activation, proliferation and reduced uptake of astroglial glutamate, being one of the main targets of pharmacological approaches to MS (115, 116). Selective knockout animals for S1P in astrocytes have reduced demyelination, axonal loss and EAE severity (117). Interestingly, we have shown that activation of S1P receptors in astrocytes improve their ability to induce maturation, neurite outgrowth and arborization of neuronal precursor cells through increase of extracellular matrix components. These results implicate S1P signaling as a key pathway in neuron-astrocyte interaction (118).

Fingolimod, a S1P receptor modulator and agonist, improves the relapse rate in RRMS, with controversial effects in the SPMS and no effect in PPMS (119–121). It is noteworthy that the CNS therapeutic effect of fingolimod in MS is mainly mediated by the inhibition of S1P receptors on astrocytes, so that astroglial S1P deletion eliminates its effect (117). It has been shown *in vitro* that reactive astrocytes derived from EAE model animals and treated with fingolimod have reduced and increased production of pro-inflammatory factors and neurotrophic factors, respectively (114, 122). Additionally, it has been shown that the anti-inflammatory mechanism of fingolimod depends on the downregulation of NFkB in astrocytes (123). On the other hand, the recovery of glutamate reuptake by astrocytes by the action of fingolimod and siponimod occurs *via* stimulation of RhoA (116). Alternatively, fingolimod also contributes to attenuation of MS through retention of leukocytes in secondary lymphoid organs (124).

Thus, the suppression of NFkB pathway must represent an important attenuation mechanism of MS. Indeed, it has been shown that inhibition of NFkB-activated pathways in astrocytes can be driven by competitive binding of the transcription factor aryl hydrocarbon receptor (AHR) by NFkB itself. AHR is highly expressed in astrocytes and is sensitive to metabolic products of the microbiota and to IFN-I (125). In the EAE model, selective deletion of the AHR in astrocytes promotes increased inflammation and worsens disease course (126).

Considering that so many signaling pathways overlap in astrocytic NFkB activation in MS, it seems that the use of drugs that suppress NFkB signaling would be more efficient than those that prevent its activation. Following this hypothesis, it is possible that stimulation of the AHR pathway, among those mentioned, has a more robust clinical effect. In line with this hypothesis, it could be considered that laquinimod, which signals *via* AHR, would be a better candidate for controlling progressive disease, where glial activity is preponderant, than other S1P analogues. Previous clinical trials showed it to be able of slowing incapacity progression in RRMS (127), although with poor relapse control (128). Nevertheless, laquinimod did not present a significant treatment effect when administered for PPMS in the ARPEGGIO study (129).

Taking into account the main hypothesis of the autoimmune origin of MS, in which the disease would be triggered by the infiltration of autoreactive T lymphocytes in the CNS, it is reasonable to consider that lymphocyte secreted factors contribute to astrocytes activation in MS. In this sense, a recent study using scRNA-seq approaches with astrocyte-specific Ribotag RNA profiling and bioinformatics analysis identified a subpopulation of reactive astrocytes expressing the GM-CSF–MAFG pathway in the EAE model and in MS (130). This astrocytic pathway is GM-CSF-driven, secreted mainly by activated T lymphocytes. In EAE, presence of GM-CSF transcripts in CNS infiltrating T lymphocytes precedes activation of the MAFG pathway in astrocytes. Elevated levels of MAFG in astrocytes, through increased methylation and repression of target genes of nuclear erythroid–derived–like factor 2 (NRF2), which includes NFkB, have been shown to result in inflammation and increased oxidative stress. In agreement with these data, inactivation of this pathway in astrocytes reduces MAFG signaling, methylation of target genes and expression of proinflammatory pathways resulting in clinical improvement in EAE (130).

NRF2-driven anti-inflammatory pathway in astrocytes has also been shown to be regulated by sirtuin-1 deacetylase (SIRT1) *in vitro* and in an EAE model (131). Thus, the selective deletion of SIRT in astrocytes in the EAE conferred an anti-inflammatory effect, promoting down regulation of pro-inflammatory factors. It also reduced recruitment of T lymphocytes, increased number of microglia and IL-10-producing monocytes, increased differentiation of oligodendrocyte progenitors and reduced demyelination. This report also showed that the majority of C3 astrocytes present in MS lesions were positive for SIRT, indicating that this is also a prominent pathway in neurotoxic astrocytes.

Although astrocytes seem to play a neurotoxic role in some demyelinating contexts, there is evidence that those cells may also provide neuroprotection through secretion of trophic factors (132). It has been shown that deletion of astrocytic BDNF generates clinical worsening and increased axonal loss in EAE (132). On the other hand, induction of BDNF production by astrocytes results in remyelination in a cuprizone model (133).

Therefore, the contribution of astrocytes for demyelinating diseases is still a subject under discussion. The most promising interventions should be those capable of converting subpopulations of neurotoxic astrocytes into an anti-inflammatory and neuroprotective state, rather than simply inhibiting their disease-associated profile. While it is clear that astrocyte signaling pathways are promise therapeutic targets for demyelinating diseases, topic is far from being closed and a deeper understanding on these pathways is required.

### 3.4 Microglia in demyelinating conditions

Microglial cells are extensively involved in the MS pathogenesis, apparently displaying a dual role of fostering inflammation, triggering synaptopathy and neurodegeneration, and sustaining the remyelination processes. These distinct roles have been associated with their heterogeneous activation profile assumed in different stages of the disease (108, 134, 135).

In active MS lesions, there is a loss of microglial homeostatic phenotype and acquisition of a reactive one (136). Transcriptome analysis revealed that in different neurodegenerative diseases, including MS, activated microglia share, in part, the same gene signature, and have been named disease-associated microglia (DAM) (137). Dependent on the TREM2-APOE pathway, microglia lose the expression of genes encoding homeostatic molecular profile (P2ry12, Tmem119, Csf1r, Hexb, Mertk, Cx3cr1) and upregulate the expression of inflammatory genes (Apoe, Itgax, Ccl2, Clec7a, Axl). TREM2-APOE pathway induces processes of migration, phagocytosis and lipid metabolism in microglia. Furthermore, activation of this pathway impairs its tolerogenic ability to suppress lymphocyte proliferation in the EAE model (137). Consistent with these findings, microglia from TREM2-deficient animals under cuprizone-induced demyelination or in models of focal lysolecithin demyelination exhibit failure in their activation, showing less proliferation, resting morphology, lower expression levels of MHC class II and iNOS, deficits in phagocytosis of myelin debris and lipid metabolism (138), persistent demyelination after chronic use of cuprizone, more aggressive reduction of oligodendrocytes and clinical worsening (138, 139).

Similarly, to active MS lesions, in active chronic lesions, mainly in the progressive phase of MS, microglial cells continue to express a reactive profile, but with a notable hallmark of phagocyted iron (140). This histopathological hallmark enables the observation of lesion rims by MRI and correlates with high levels of serum neurofilament light chain, therefore with axonal injury, and with disease severity (141, 142). A recent study revealed the existence of two main reactive microglial phenotypes on the edge of the active chronic lesions: microglia-inflamed MS (MIMS)-foamy, with a genetics signature similar to DAM, also being associated with myelin phagocytosis and clearance; and MIMS-iron, with pro-inflammatory molecular features, including increased expression of MHC class II, ferritin complex, FcY receptor for immunoglobulin, and complement C1 complex (C1QA and C1QB) expressing genes for ribosomal proteins, indicating a role in antigen presentation and perpetuation of inflammation (108).

In general, evidence indicate that in the onset of MS, the immune response of microglia is less relevant than that of peripheral macrophages for the inflammatory process (135, 143). In fact, in the CNS infiltrated peripheral myeloid cells, inflammatory signaling (pSTAT3) is significantly higher than in the CNS resident ones from the beginning to the peak of EAE (144). This same study showed that the inflammatory signaling pathway, NFkB, in microglia only increases in the chronic phase of EAE. Furthermore, MHC class II-dependent microglia antigen presentation seems dispensable for disease establishment and progression, as observed in EAE and cuprizone models (145), corroborating to a less prominent role of these cells, at least, in triggering inflammation. In turn, microglial cells contribute to myelin clearance at the onset of lesions, being indispensable for the process of remyelination and tissue repair (146).

In fact, a study using CNS samples from EAE demonstrated that microglia with differential expression of myeloid activation marker (MHC classII, CD86+), in addition to TREM2+, progressively increase from the asymptomatic phase to the peak of EAE (144). Although it has been shown that different myelin lipids directly activate the TREM2 pathway (139), the hypothesis of a transition from MIMS-foam to MIMS-iron after myelin phagocytosis has not yet been investigated, but it is possible that these signatures correspond to different phases of activation from the same cell. In this sense, it has been shown that the myelin inhibitory protein Nogo-A is able to induce a pro-inflammatory profile in microglia dependent on NFkB activation (147). This event may represent a possible mechanism by which myelin phagocytosis results in a pro-inflammatory response and would explain the acquisition of the inflammatory role of microglia in later stages of the lesion.

Activated microglia in MS and EAE are associated with the production of several pro-inflammatory cytokines (IL-6, IL-1β, IL-18, IL-12, IL-23, TNF-α) and chemokines (CCL2, CCL3, CCL4, CCL5, CCL7, CCL12) (135). *In vivo* and *ex vivo* studies in animal models of MS have shown that the pro-inflammatory cytokines IL-1β, TNF-α and IL-18 are involved in the synaptic loss of the disease, which may occur independently of demyelination and neuronal degeneration (45, 148). An enzymatic complex named NRLP3 inflammasome is involved in the conversion of immature forms of IL-1β, and IL-18 into active ones, being a component found in microglia and involved in CNS diseases (149). NRLP3 inflammasome inhibition reverses hippocampal synaptic loss and memory deficits of the late phase of EAE (148). In microglia, its inhibition may be mediated by NF-κB regulatory protein A20 (135). Thus, the use of A20-deficient mice selectively on microglia generates an EAE model of early onset and aggravated pathogenesis. This effect is generated through hyperactivation of the inflammasome, resulting in increased IL-1β secretion and CNS inflammation. Increased levels of the A20-NLRP3 pathway were also found to be increased in the lesions and CSF of MS patients.

One of the mechanisms of synaptic clearance in MS seems to involve phagocytosis of the synapse by microglia, previously opsonized by complement protein. Complement proteins C1q and C3 are increased in MS, and can be seen at synapses located within HLA-positive cell processes and in lysosomes, indicating opsonization and engulfment by microglia (150). Confirming this idea, viral vector reduction of C3 at synapses in the visual system of non-human primate and mouse animal models of MS reduced microglial phagocytosis and protected visual function (151).

The presence of activated microglia/macrophages has been demonstrated in demyelinating lesions close to the location of neurotoxic astrocytes expressing C3 (152). This astroglial phenotype can be induced through the secretion of IL-1β, TNF-α and C1q by microglia. This was one of the first works to demonstrate that crosstalk between astrocytes and microglia is an important regulator of the glial reactivity state.

Complex mechanisms of communication between astro- and microglia have also been described to occur in other neurodegenerative diseases and their understanding lays groundwork for new targets for therapeutic strategies. Absinta and collaborators (108) revealed that in the edge of the chronic active MS lesion, but not in the chronic inactive ones or in the core of the lesion, MIMS and AIMS correspond to a central hub of interactions that connect with other glial populations and immune (108). They identified genes of the complement system as important mediators of the crosstalk between astro- and microglia. While MIMS-iron upregulates transcripts to genes codifying to C1QA, C1QB, C1QC, and CFD, AIMS upregulates to activators of the C1Q complex, C1Q receptors, and C3. It also demonstrated that in patients with a higher frequency of lesions rims (>4) there are more risk variants of genes for complement system, indicating an important correlation between the alteration of this system and more aggressive forms of the disease. Furthermore, the EAE mouse with selective microglial C1q deletion or treated with C1q inhibitor showed reduced levels of microgliosis and signature markers of DAM and MIMS, although without improvement of the clinical score or change in the disease onset date. Thus, microglial C1q was identified as the mediator of MIMS activation and must be important for the perpetuation of inflammation in active chronic lesions.

Recently, by using the SPEAC technique with co-culture of cells in drops and CRISPR-Cas induced genetic manipulation, Wheeler and collaborators (153) identified the amphegulin–IL33-ST2 pathway as another communication mechanism between microglia and astrocytes, relevant to the pathogenesis of MS. This signaling has been shown to limit the NFkB-mediated pro-inflammatory response in astrocytes from EAE.

Together, those results show that understanding the interaction between microglia and astrocytes in demyelination and, more specifically, in the pathogenesis of MS, should provide key clues for new DMTs, that are certainly missed by looking at those subpopulation of glial cells individually.

## 4 Current therapeutic strategies and glial cells

Currently, the gold standard of MS treatment varies with the course of the disease but involves both pharmacological treatment and multidisciplinary rehabilitation methods. In the case of NMOSD, only recently treatments specifically approved for AQP4ab positive patients have become available. For MOGAD, there is still no largely established treatment regimen. For further reference, a summary of drugs mostly used in those diseases can be found in **Table 2**. As no curative treatment exists for either phenotype, the search for disease-modifying therapies is still crucial.

**Table 2:** Available long-term pharmacotherapy options for CNS IDDs.

Medication	Brand name	Pharmacologic class	Established use	Administration	Action mechanism
Alemtuzumab	Lemtrada	Monoclonal antibody against CD52.	Treatment of RRMS.	Intravenous infusion.	Induces cell death in CD52+ lymphocyte subpopulations.
Azathioprine	Imuran, Azasan	Purine analogue.	Off-label treatment of NMOSD.	Oral administration.	Blocks purine synthesis through 6-thioguanine metabolites.
Cladribine	Mavenclad	Purine analogue.	Treatment of RRMS.	Oral administration.	Disrupts DNA synthesis in B and T lymphocytes.
Dimethyl fumarate	Tecfidera	Fumaric acid derivative.	Treatment of RRMS.	Oral administration.	Modulates response to oxidative stress by activation of Nrf2 pathway.
Diroximel fumarate	Vumerity	Fumaric acid derivative.	Treatment of RRMS.	Oral administration.	Modulates response to oxidative stress by activation of Nrf2 pathway.
Eculizumab	Soliris	Humanized monoclonal antibody against C5.	Treatment of AQP4ab serum-positive NMOSD.	Intravenous infusion.	Prevents cleavage of C5 and formation of membrane attack complex.
Fingolimod	Gilenya, Tascenso ODT	S1P receptor modulator.	Treatment of RRMS.	Oral administration.	Unselectively binds to S1P receptors; induces lymphocyte sequestration.
Glatiramer acetate	Copaxone, Glatopa	Aminoacid polymer.	Treatment of RRMS.	Subcutaneous injection.	Interferes with antigen-presenting of immune cells by mimicry of myelin antigens.
Inebilizumab	Uplizna	Monoclonal antibody against CD19.	Treatment of AQP4ab serum-positive NMOSD.	Intravenous infusion.	Induces cell death in CD19+ B cells.
Interferon beta-1a	Avonex, Rebif	Interferon.	Treatment of RRMS.	Intramuscular (Avonex) and subcutaneous (Rebif) injections.	Enhances immune cell activity; down regulates antigen presentation.
Interferon beta-1b	Betaseron, Extavia	Interferon.	Treatment of RRMS.	Subcutaneous injection.	Downregulates antigen presentation; reduces lymphocyte trafficking and proinflammatory cytokine release.
Mitoxantrone	Novantrone	Anthracenedione.	Treatment of RRMS and SPMS.	Intravenous infusion.	Decreases leukocyte replication by inhibiting DNA topoisomerase II.
Monomethyl fumarate	Bafiertam	Fumaric acid derivative.	Treatment of RRMS.	Oral administration.	Modulates response to oxidative stress by activation of Nrf2 pathway.
Mycophenolate mofetil	Cellcept	Purine synthesis inhibitor.	Off-label treatment of NMOSD and MOGAD.	Oral administration; intravenous infusion.	Inhibits IMPDH, impeding guanosine synthesis; suppresses mTOR and STAT5 in CD4+ cells.
Natalizumab	Tysabri	Monoclonal antibody against alpha-4 integrin.	Treatment of RRMS.	Intravenous infusion.	Blocks T cell migration into CNS by impeding integrin association with vascular receptors.
Ocrelizumab	Ocrevus	Humanized monoclonal antibody against CD20.	Treatment of RRMS and PPMS.	Intravenous infusion.	Induces cell death in CD20+ B cells by antibody-dependent cellular cytotoxicity.
Ofatumumab	Kesimpta	Human monoclonal antibody against CD20.	Treatment of RRMS.	Subcutaneous injection.	Induces cell death in CD20+ B cells by antibody-dependent cellular cytotoxicity.
Ozanimod	Zeposia	S1P receptor modulator.	Treatment of RRMS.	Oral administration.	Selectively binds to S1PR1, with low binding to S1PR5; induces lymphocyte sequestration.
Peginterferon beta-1a	Plegridy	Interferon.	Treatment of RRMS.	Subcutaneous injection.	Enhances immune cell activity; down regulates antigen presentation.
Prednisone	Rayos, Winpred	Systemic corticosteroid.	Off-label treatment of NMOSD and MOGAD; RRMS relapses.	Oral administration.	Suppresses migration of polymorphonuclear leukocytes; inhibits lymphocyte activity.
Rituximab	Rituxan	Chimeric monoclonal antibody against CD20.	Off-label treatment for RRMS, NMOSD and MOGAD.	Intravenous infusion.	Induces cell death in CD20+ B cells by antibody-dependent cellular cytotoxicity.
Satralizumab	Enspryng	Humanized monoclonal antibody against IL-6 receptor.	Treatment of AQP4ab serum-positive NMOSD.	Subcutaneous injection.	Binds to IL-6 receptor preventing its pathway signaling and down regulating IL-6-dependent inflammatory response.
Siponimod	Mayzent	S1P receptor modulator.	Treatment of RRMS and SPMS.	Oral administration.	Selectively binds to S1PR1 and S1PR5; induces lymphocyte sequestration.
Teriflunomide	Aubagio	Pyrimidine synthesis inhibitor.	Treatment of RRMS.	Oral administration.	Blocks dihydroorotate dehydrogenase activity, impeding pyrimidine synthesis.
Tocilizumab	Actemra	Humanized monoclonal antibody against IL-6 receptor.	Off-label treatment for MOGAD.	Subcutaneous injection; intravenous infusion.	Binds to IL-6 receptor preventing its pathway signaling and down regulating IL-6-dependent inflammatory response.
Ublituximab	Briumvi	Chimeric monoclonal antibody against CD20.	Treatment of RRMS.	Intravenous infusion.	Induces cell death in CD20+ B cells by complement- and antibody-dependent cellular cytotoxicity.

Drug information presented accordingly to that provided by manufacturers.

Liu and colleagues (154) evaluated 21 recent studies regarding DMTs for RRMS, using as parameters adverse effects, risk for relapses and study consistency. Among the evaluated interventions, the monoclonal antibodies ofatumumab, alemtuzumab, ocrelizumab and natalizumab showed higher efficiency. However, these therapies are limited, needing intravenous administration in a hospital setting as a prerequisite.

Considering that astro- and microglia show typical behavior in demyelination and may sustain neurodegeneration and neuroinflammation, those cells are suitable candidates as therapeutic targets. As of 2023, no DMT had been designed or approved to counter neurodegeneration and glial activity as a primary mechanism. This does not mean, however, that currently approved drugs do not affect glia directly.

Oral immunosuppressors such as fingolimod and siponimod have proved to be highly efficient in treatment of MS relapses. Among their advantages are the possibility of treating patients in an outpatient scenario and giving them greater autonomy over disease and treatment. When comparing natalizumab and fingolimod, though, it was found that while the former had a 1.9% relapse rate in follow-up, the later was as high as 22.3%. Furthermore, fingolimod has been associated with a higher rate of MRI lesions in treatment outcome (155). Another burden is that fingolimod use is associated with a higher infection rate in MS patients (16% higher than placebo), but there’s no convincing data comparing it with other drugs or drug candidates in this regard (156).

In an extensive literature review, Kim De Kleijin evaluated the molecular pathways activated by the different FDA-approved drugs for MS, showing NFκB signaling as a common denominator of their effects in both microglia and astrocytes (157). Fingolimod, a drug which first arose due its role on lymphocyte migration, showed the ability to induce angiogenesis by inducing a M2 phenotype in microglia through S1PR1 modulation (158) and STAT3 signaling (159). In another study, fingolimod also improved white matter tract integrity, accessed by diffusion tensor imaging (160). Fingolimod is the prototype molecule in a class of sphingosine-1-phosphate (S1P) analogues, interacting with the different S1P receptors (S1PR1-5), a class of G-coupled receptors. As such, fingolimod is unselective as to which receptors it binds, probably spoiling its effects on the CNS when compared to newer drugs. As discussed previously, a tentative example of selective effect would be modulation of S1PR2 in astrocytes, which seems to be linked to glutamate recycling and mitochondrial metabolism (116), both promising mechanisms to ameliorate excitotoxic synaptopathy.

One currently available selective S1P agonist exists in the form of siponimod, which binds to S1PR1 and S1PR5. In tests with animal models, it was able to induce remyelination and proliferation of mature oligodendrocytes, an effect attributed to the induction of a regeneration-supporting microglia phenotype, in a dose-dependent manner (161), associated with S1PR1 activity. In human astrocytes, S1PR1 activation by siponimod improved Ca^2+^ signaling and receptor internalization (162), while it inhibited NFκB by nuclear translocation of Nrf2, a regulator of antioxidant response (64). Siponimod was also able to rescue the expression of glutamate transporters EEAT1 and EEAT2 when under an inflammatory insult. Further, when using conditioned medium from treated astrocytes, siponimod also inhibited axonal degeneration *in vitro.* In both cell types the drug attenuated reactivity to lipopolysaccharide and interleukins (162). Findings such as these may suggest a neuroprotective role for siponimod in the CNS, by countering MIMS and AIMS development, and justify its feasibility as a therapeutic option in SPMS (163). Not surprisingly, employment of siponimod in SPMS has been observed to slow gray matter atrophy and reduce peripheral levels of neurofilament light chain (164). Ponesimod, another S1P analogue exclusively binding to S1PR1, showed high tropism to astrocytes, causing a long-term antagonism to receptor activity. In this context, pro-inflammatory genes were downregulated in the astroglia and demyelination was partially prevented in a murine cuprizone-induced demyelination model (165). When tested in a model of a non-demyelinating disease (subarachnoid hemorrhage), siponimod prevented neuronal death by suppressing A1 polarization in astrocytes, further showing its anti-inflammatory properties in CNS cells (147).

A non-S1P-related drug which also appears to protect glial homeostasis is dimethyl fumarate (DMF). DMF is an immunomodulator used in RRMS and is an ester of fumaric acid. Its effects are thought to be associated with oxidative stress and downregulation of aerobic glycolysis (166). In mouse microglia, DMF impairs NLRP3 inflammasome activation, an effect presented both *in vivo* and *in vitro* (167). The affected domains included cell survival, cytokine release and oxidative stress. DMF also reduced cell motility and migration, dependent on cytoskeleton rearrangement, and the uptake of iron content (168). As in the case of siponimod, the results were related to NFκB and Nrf2 pathways. There remains doubt whether glycolysis is a relevant mechanism in this situation, as another study demonstrated this process was only regulated by DMF in microglia from females (169). Therefore, we can say mechanistic studies of this drug in microglia are still limited. We can also see a direct effect on oligodendrocytes, preserving their lipid metabolism, which may favor myelin homeostasis (170). In a model of EAE, the reduction of C3 deposition by DMF treatment also reduced the number of reactive astrocytes present (171). This probably includes a direct interaction between the drug and astroglia, as the *in vitro* treatment of human cells with DMF was seen to reduce secretion of such factors as IL-6, CXCL10, and CCL2 (172). The effect on astrocytes may happen independently of Nrf2, since cofilin-1, tubulin and collapsin response mediator protein 2 (CRMP2) activity was seen to be mediated by DMF and reduced cytoskeletal remodeling (173). Other mechanisms possibly involved are glutathione and heme oxygenase-1 (174) as well as histone deacetylases expression (175). Both of those, however, still involve Nrf2 activity.

Despite the cumulative effects on the various glia, DMF has no identified purpose in the treatment of progressive disease. As a matter of fact, although DMF reduced iron content in chronically active lesions (177), a meta-analysis concluded that there was little evidence for ameliorating disease progression even on RRMS (176). Whether this means DMF leads to favorable subclinical outcomes and how much those outcomes are relevant needs further investigation.

Another biological process in which the role of glial cells is preponderant and central to MS and other IDDs, is demyelination. Inducing remyelination is thus considered pivotal to novel therapeutic strategies. Since this is such a complex and finely-tuned system, we still lack an efficient drug designed for this. As previously discussed, exploratory studies targeting myelin proteins, myelin turnover and oligodendrocyte maturation may prove fruitful in that regard.

Alternatively, neuroprotective strategies for long-term disease are also a growing field. However, the variety of candidates (RNAs, small molecules, lipids, inorganic compounds, dietary supplements) suffers from the still incipient clinical investigation methods, which represent only 8% of the recently published papers on the matter (178). Thus, no tentative strategy seems close to large-scale application. In this field, the most promising candidate drug seems to be the class of Bruton Tyrosine Kinase (BTK) Inhibitors (BTKi).

BTK translocation is linked with NFkB and NFAT activation (179). Although this class was initially proposed as B-cell targeting therapy without cell-line depletion, as BTK plays a role in B-cell receptor activation, this type of molecule has a role in development of myeloid cell line and may also modulate microglial NFkB pathway (180). Particularly, BTK seems to play a significant role in microglial proliferation when these cells are exposed to anti-MOG antibodies (181). An *in vitro* study with ibrutinib, another BTKi, revealed that treatment with the inhibitor was capable of reducing the effect of LPS-induced inflammation on microglia, but not astrocytes. It reduced the levels of pSTAT-3 and p-AKT in those cells, resulting in lower expression of pro inflammatory cytokines. In the same study, wild type mice injected with ibrutinib also showed reduced micro- and astroglial activation. As ibrutinib had no direct effect upon astrocytes, this may be explained by further glial cell crosstalk (182). Alternatively, BTKi may act through direct metabolic modulation, since it has been shown that BTKi treatment reduced mitochondrial activity of B cells and B cell antigen-presenting activity (183).

Accordingly, diverse BTKi are currently under study for both RRMS and PPMS in ongoing clinical trials, the most advanced ones being evobrutinib and tolebrutinib (184). Evobrutinib, an oral administration selective BTKi, has lowered the number of gadolinium-enhanced lesions in a phase II clinical trial, when compared with DMF. On the other hand, it did not show any superiority in clinical parameters, both in the annualized relapse rate and disability progression (185). Further studies to identify benefits of exploring those pathways, particularly in PPMS, are still needed.

## 5 Concluding remarks

As demonstrated, the main glial cells in CNS are largely involved in the pathological processes implicated in the IDDs, and particularly in the different courses of MS. As in the case of NMOSD, previously seen as another MS phenotype and now largely understood as an astrocytopathy, better comprehension of the mechanisms by which glia relate to neuroinflammatory diseases may arise and reveal completely new aspects of those disorders. In the case of MS, while axonal and neuronal destruction is the ultimate fate and cause of disability in disease, glia is present in inflammation (microglia), myelin loss and tentative regeneration (oligodendrocyte) and synaptopathy itself (astrocytes). Following all this, myelin inhibitory proteins, such as Nogo-A, and inflammasome components may appear as possible links between the facets of these disorders.

Still regarding CNS disease and considering the growing importance of glial cells to the understanding of nervous system function and homeostasis, we suggest that these cells are at the center of pathological processes within the brain, with demyelinating diseases representing an ideal example of global disruption of glial function in disease. Thus, we consider that it will become ever more pertinent to see glia less as support cells secondarily affected by disease, and more as central players in the physiopathology, at the hub of disease.

In this review we sought to report the most recent breakthroughs regarding glial cells and inflammatory demyelinating diseases of the CNS. For the scope of the article and extensiveness of the field, it is impossible to cover in all its aspects; for this we decided to focus on the more evident case of MS. We understand these diseases cannot be fully grasped without the correct assumption of the molecular and cellular interactions present in their pathogenesis. To that end, the text highlights the neurodegenerative aspect of demyelination and tries to employ a “non-neuroncentric” perspective on the matter. Accordingly, we have attempted to propose a way that the different cell types in the brain may correlate demyelination, inflammation and neurodegeneration, exemplifying for this the Nogo-A and NFkB pathways. We hope more studies may aggregate firmer evidence for this, as new approaches to the problem of MS and IDDs in general remain necessary as ever.

## Author contributions

VC was the primary author. He searched for and selected bibliography, wrote mostly of the sections and made all figures and tables. SE-SA reviewed the full text and gave insights on the discussion of animal models. She wrote text which became part of section 3. SA-L reviewed the full text and gave insights on clinical features of diseases, diagnostic criteria and therapeutics. She wrote text which became part of section 1. FG is the senior author. She defined the article scope, selected bibliography, reviewed the full text and supervised the confection of the whole manuscript. All authors contributed to the article and approved thesubmitted version.
